# Expression of DNA Methyltransferase 3B Isoforms Is Associated with DNA Satellite 2 Hypomethylation and Clinical Prognosis in Advanced High-Grade Serous Ovarian Carcinoma

**DOI:** 10.3390/ijms232112759

**Published:** 2022-10-22

**Authors:** Victor M. Del Castillo Falconi, José Díaz-Chávez, Karla Torres-Arciga, Fernando Luna-Maldonado, Adriana A. Gudiño-Gomez, Abraham Pedroza-Torres, Clementina Castro-Hernández, David Cantú de León, Luis A. Herrera

**Affiliations:** 1 Instituto Nacional de Cancerología (INCAN), Unidad de Investigación Biomédica en Cáncer, Instituto de Investigaciones Biomédicas, UNAM, Avenida San Fernando No. 22, Sección XVI Tlalpan, Ciudad de México 14080, Mexico; 2 Instituto Nacional de Medicina Genómica, Periferico Sur 4809, Arenal Tepepan, Tlalpan, Ciudad de México 14610, Mexico; 3 Cátedras CONACYT—Instituto Nacional de Cancerología, Ciudad de México 14610, Mexico

**Keywords:** ovarian cancer, DNMT3B isoforms, histological grade, biomarkers, cancer epigenetics, DNA methylation

## Abstract

Alterations in DNA methylation are critical for the carcinogenesis of ovarian tumors, especially ovarian carcinoma (OC). DNMT3B, a de novo DNA methyltransferase (DNMT), encodes for fifteen spliced protein products or isoforms. DNMT3B isoforms lack exons for the catalytic domain, with functional consequences on catalytic activity. Abnormal expression of DNMT3B isoforms is frequently observed in several types of cancer, such as breast, lung, kidney, gastric, liver, skin, leukemia, and sarcoma. However, the expression patterns and consequences of DNMT3B isoforms in OC are unknown. In this study, we analyzed each DNMT and DNMT3B isoforms expression by qPCR in 63 OC samples and their association with disease-free survival (DFS), overall survival (OS), and tumor progression. We included OC patients with the main histological subtypes of EOC and patients in all the disease stages and found that DNMTs were overexpressed in advanced stages (*p*-value < 0.05) and high-grade OC (*p*-value < 0.05). Remarkably, we found DNMT3B1 overexpression in advanced stages (*p*-value = 0.0251) and high-grade serous ovarian carcinoma (HGSOC) (*p*-value = 0.0313), and DNMT3B3 was overexpressed in advanced stages (*p*-value = 0.0098) and high-grade (*p*-value = 0.0004) serous ovarian carcinoma (SOC). Finally, we observed that overexpression of DNMT3B isoforms was associated with poor prognosis in OC and SOC. DNMT3B3 was also associated with FDS (*p*-value = 0.017) and OS (*p*-value = 0.038) in SOC patients. In addition, the ovarian carcinoma cell lines OVCAR3 and SKOV3 also overexpress DNMT3B3. Interestingly, exogenous overexpression of DNMT3B3 in OVCAR3 causes demethylation of satellite 2 sequences in the pericentromeric region. In summary, our results suggest that DNMT3B3 expression is altered in OC.

## 1. Introduction

Ovarian cancer (OC) is the most lethal cancer of the female genital tract. In the United States, OC is responsible for more cancer-related deaths than all other gynecological tumors [1]. OC is a heterogeneous histological cancer. There is non-epithelial cancer, an uncommon group comprising germinal and stromal tissues; however, the most frequent tumor is epithelial ovarian cancer, which comprises more than 90% of all OC cases. The subtypes of epithelial ovarian cancer (EOC) are serous ovarian carcinoma (SOC), endometrioid carcinoma (EC), mucinous carcinoma (MC), clear cells carcinoma (CCC), and mixed carcinoma [2,3].

Alterations in DNA methylation have a role in the development of cancer cells and other diseases [4,5]. In cancer, DNA methylation has been associated with clinical diagnosis and prognosis. Specifically, in EOC, DNA methylation has been associated with nuclear size, proliferation index, and chromosomal ploidy [6,7,8,9]. In EOC, it has been reported that DNA is globally hypomethylated [10], but the gene promoters of tumor suppressors such as BRCA1 [11], RASSFF1A [12], CDKN2A [13], and p16 [14], among others, are hypermethylated.

DNA methylation is catalyzed by DNA methyltransferases (DNMTs), wherein DNMT1 is essential in maintaining DNA methylation patterns through the cell cycle, whereas DNMT3A and DNMT3B are in charge of methylation patterns’ establishment in the genome [15,16]. Alternative splicing regulates DNMT1 [17], DNMT3A [18], and DNMT3B isoforms’ expression [19]. DNMT1 and DNMT3A isoforms are catalytically active, and DNMT3B encodes several catalytically inactive isoforms. It has been reported that DNMT3B has fifteen protein products resulting from alternative splicing, including seven DNMT3Bs, seven ΔDNMT3B, and one DNMT3B3-like product [20,21,22,23,24]. DNMT3B isoforms lack exons that encode for the catalytic domain, causing their loss or reduction of catalytic activity [25,26,27]. The consequences of some DNMT3B isoforms’ overexpression have been studied in several models of cancer cell lines, where DNMT3B4 and DNMT3B7 isoforms have activities such as growth suppressors, differentiation, and altering DNA methylation patterns [28,29,30]. The overexpression of ΔDNMT3B4 in a transgenic mouse model induces lung hyperplasia and alters DNA methylation patterns [20]. Overexpression of DNMT3B isoforms has also been found in embryonic development [19,31,32].

The overexpression of DNMT3B isoforms has been reported in several types of cancer cells; for example, DNMT3B3 and DNMT3B4 in gastric cancer [33], DNMT3B4 in the liver and clear cells renal cancers [33,34,35], DNMT3B7 in breast cancer [36,37], and ΔDNMT3B4 in lung cancer [20]; however, the expression patterns and consequences of DNMT3B isoforms’ overexpression in EOC are still unknown. Therefore, this study aimed to analyze the expression patterns of DNMT3B isoforms and their association with clinical variables such as tumor grade, disease stage, and prognostic value in EOC. We used real-time reverse transcription PCR assays to quantify the expression of DNMT3B isoforms in 63 epithelial EOC tumor samples. We found that overexpression of DNMT3B3 was associated with poor disease-free survival and overall survival of epithelial ovarian cancer patients. We also observed that DNMT3B, DNMT3B3, and DNMT3B3Δ5 have higher levels in high-grade and advanced stages compared with low-grade and early stages, respectively. In summary, these results suggest that DNMT3B3 overexpression could be important in EOC progression and aggressiveness.

## 2. Results

### 2.1. DNA Methyltransferases Expression in Ovarian Carcinoma

**Figure 1:**
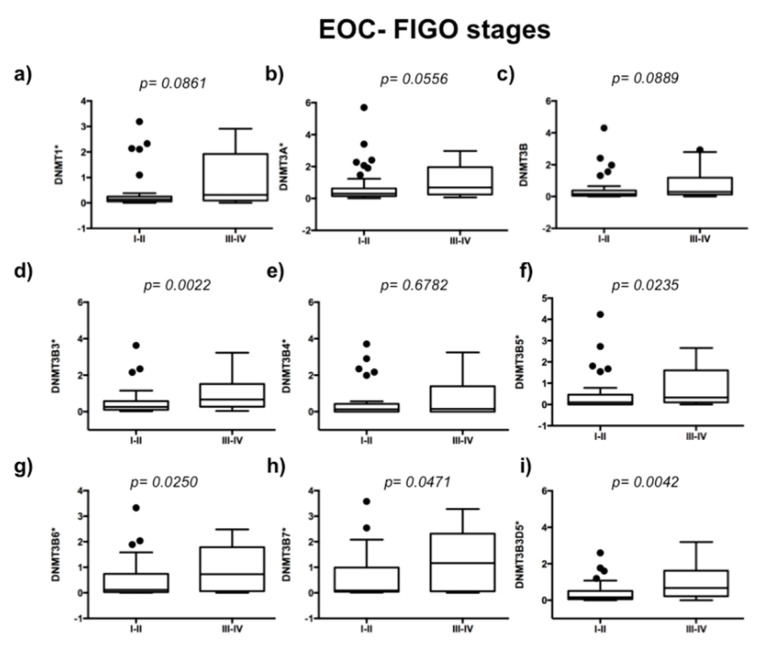
Overexpression of DNMT3B3, DNMT3B5, DNMT3B6, DNMT3B7, and DNMT3B3Δ5 according to the clinical stage of epithelial ovarian cancer (EOC). DNMT isoforms’ expression in stages I–II (early disease stages) (*n* = 36) versus stages III–IV (late disease stages) (*n* = 27). (**a**) DNMT1, (**b**) DNMT3A, (**c**) DNMT3B, (**d**) DNMT3B3, (**e**) DNMT3B4, (**f**) DNMT3B5, (**g**) DNMT3B6, (**h**) DNMT3B7, and (**i**) DNMT3B3Δ5. Assays were normalized with GAPDH expression in each case and ten controls of the normal ovary. The Mann–Whitney U test was used. * 1 + log-transformed.

**Figure 2:**
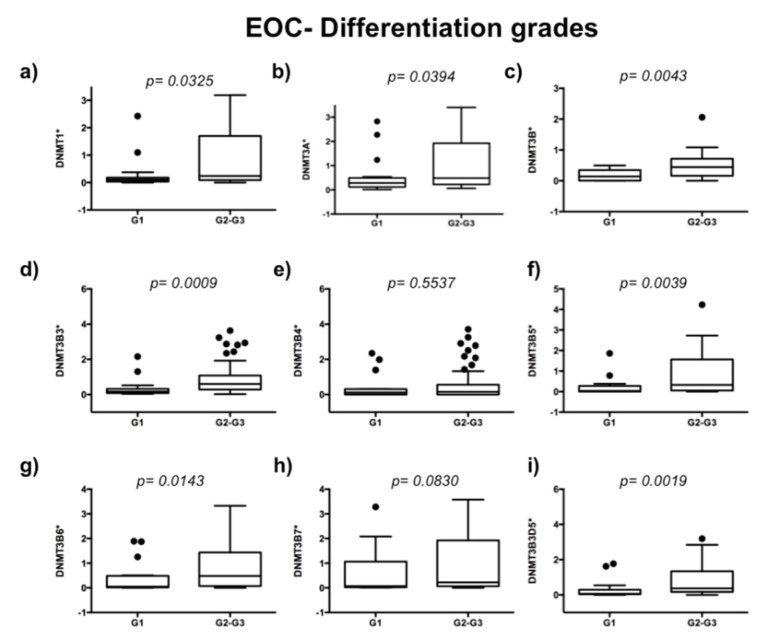
Expression of DNMT1, DNMT3A, DNMT3B3, DNMT3B5, DNMT3B6, and DNMT3B3Δ5 according to histological grade. DNMTs’ expression in low-grade (n = 13) versus high-grade (n = 50). (**a**) DNMT1, (**b**) DNMT3A, (**c**) DNMT3B, (**d**) DNMT3B3, (**e**) DNMT3B4, (**f**) DNMT3B5, (**g**) DNMT3B6, (**h**) DNMT3B7, and (**i**) DNMT3B3Δ5. Assays were normalized with GAPDH expression in each case and ten controls of the normal ovary. Mann–Whitney U test was used. * 1 + log-transformed. Tumor grade: G1 (low grade), G2–G3 (medium–high grade).

To evaluate the expression of DNMTs in 63 EOC samples, we used 10 ovarian disease-free tissue samples as controls of DNMTs’ basal expression. We analyzed if DNMTs are related to the ovarian carcinoma stage of the disease. We observed higher expression of DNMT3B3 (*p* = 0.0022), DNMT3B5 (*p* = 0.0235), DNMT3B6 (*p* = 0.0250), DNMT3B7 (*p* = 0.0471), and DNMT3B3Δ5 (*p* = 0.0042) in an advanced stage of ovarian carcinoma (Figure 1d,f–i). In contrast, we did not observe significant differences in the expression of DNMT1 (*p* = 0.0861), DNMT3A (*p* = 0.0556), DNMT3B1 (*p* = 0.889), and DNMT3B4 (*p* = 0.6782) (Figure 1a–c,e). Then, we compared DNMTs’ expression from high-grade tumors (medium- and high-grade endometrioid cancer cells, clear cancer cells, high-grade serous ovarian carcinomas, and mixed ovarian cancers) versus low-grade EOC samples (low-grade endometrioid, mucinous cancer cells, and low-grade serous ovarian carcinomas) (Figure 2). We found higher expression of DNMT1 (*p* = 0.0325), DNMT3A (*p* = 0.0394), DNMT3B1 (*p* = 0.0068), DNMT3B3 (*p* = 0.0171), DNMT3B5 (*p* = 0.0039), DNMT3B6 (*p* = 0.0143), and DNMT3B3Δ5 (*p* = 0.0148) in high-grade ovarian carcinoma (Figure 2a–d,f,g,i). In contrast, we did not find significant differences in the expression of DNMT3B4 (*p* = 0.5537) and DNMT3B7 (*p* = 0.0830) (Figure 2e,h).

### 2.2. DNMT3B1 and DNMT3B3 Are Overexpressed in High-Grade and Advanced Stages of Serous Ovarian Carcinoma

**Figure 3:**
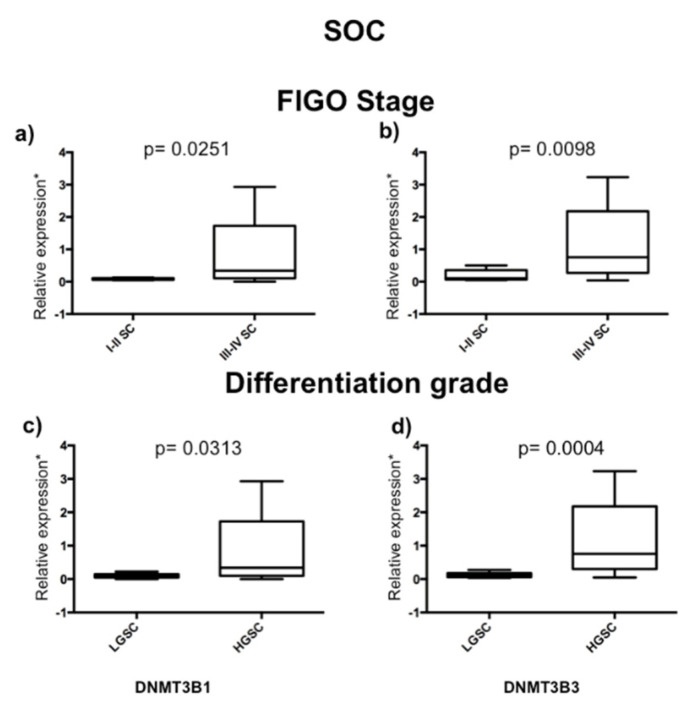
DNMT3B1 and DNMT3B3 expression according to grade and clinical stages in serous ovarian carcinoma (SOC). DNMT3B isoforms’ expression in serous ovarian carcinoma (n = 27): LGSOC (low-grade serous carcinoma) (n = 6) versus HGSOC (high-grade ovarian carcinoma) (n = 21) and stages I–II (early disease stages) (n = 6) versus stages III–IV (advanced stages) (n = 21). DNMT3B1: (**a**) grade and (**c**) stage, and DNMT3B3: (**b**) grade and (**d**) stage. Assays were normalized with GAPDH expression in each case and ten controls of the normal ovary. Mann–Whitney U test was used. * 1 + log-transformed.

**Figure 4:**
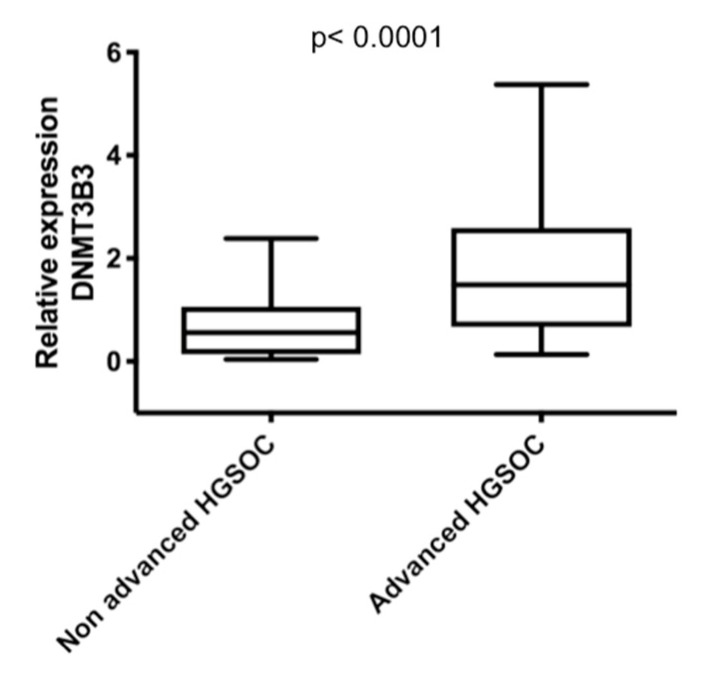
DNMT3B3 overexpression validated in advanced stages of high-grade serous ovarian carcinoma (HGSOC). DNMT3B3 isoform overexpression in two arrays of cDNA rapid cDNA panels: II and III, of ovarian carcinoma (TissueScan Ovarian Cancer cDNA arrays II and III) (OriGene Technologies, Rockville MD) (cat. no. HORT102, and 103) (n = 82). Non-advanced HGSOC (n = 23: mucinous, low-grade serous ovarian carcinoma, endometrioid, carcinoma clear cells, and stages I–II of HGSOC) versus advanced HGSOC (n = 59: stages III–IV of HGSOC). Assays were normalized with GAPDH expression in each case and ten controls of the normal ovary. Mann–Whitney U test was used.

Then, we analyzed if DNMT1, 3A, 3B1, and DNMT3B3, 3B4, 3B5, 3B6, 3B7, and 3B3Δ5 isoforms’ expression was associated with the stage of disease and tumor grade in serous ovarian carcinoma (SOC). We only found differences in DNMT3B1 and 3B3 expression from advanced stages (stages III and IV) (n = 27) with early stages (stages I and II) (n = 6) of serous ovarian carcinoma, and high-grade (HGSOC) (n = 27) with low-grade (LGSOC) (n = 6) (Figure 1). We found significant overexpression of DNMT3B1 and DNMT3B3 in HGSOC and advanced stages compared with LGSOC and early stages of SOC (Figure 3). To validate that DNMT3B3 is overexpressed in advanced stages of HGSOC, we used two arrays of cDNA rapid cDNA panels: II and III, of ovarian carcinoma (TissueScan Ovarian Cancer cDNA arrays II and III) (OriGene Technologies, Rockville, MD, USA) (cat. no. HORT102 and 103), and both had 14 ovaries without cancer and 82 ovarian carcinoma samples of cDNA to be analyzed (Figure 4).

### 2.3. DNMT3B3 Overexpression Is Associated with Poor Prognosis in Serous Ovarian Carcinoma

**Figure 5:**
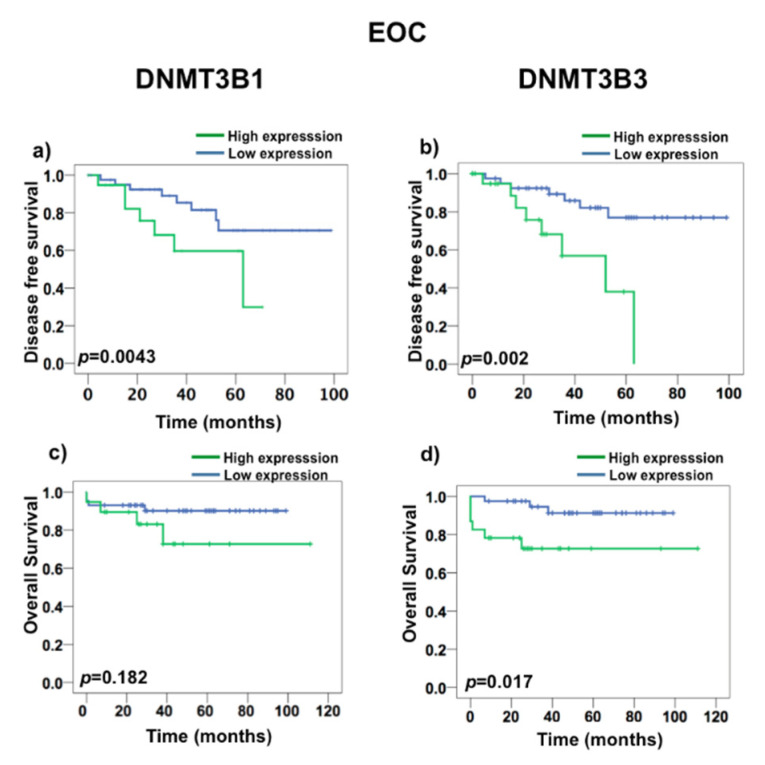
DNMT3B3 overexpression is associated with disease-free survival in ovarian carcinoma (EOC). DNMT3B1 expression in Kaplan–Meyer survival analysis in EOC: (**a**) DFS and (**c**) OS, and DNMT3B3 in EOC: (**b**) DFS and (**d**) OS. The log rank test was used to calculate the significant differences among overexpression (n = 46) versus no overexpression (n = 23) groups.

**Figure 6:**
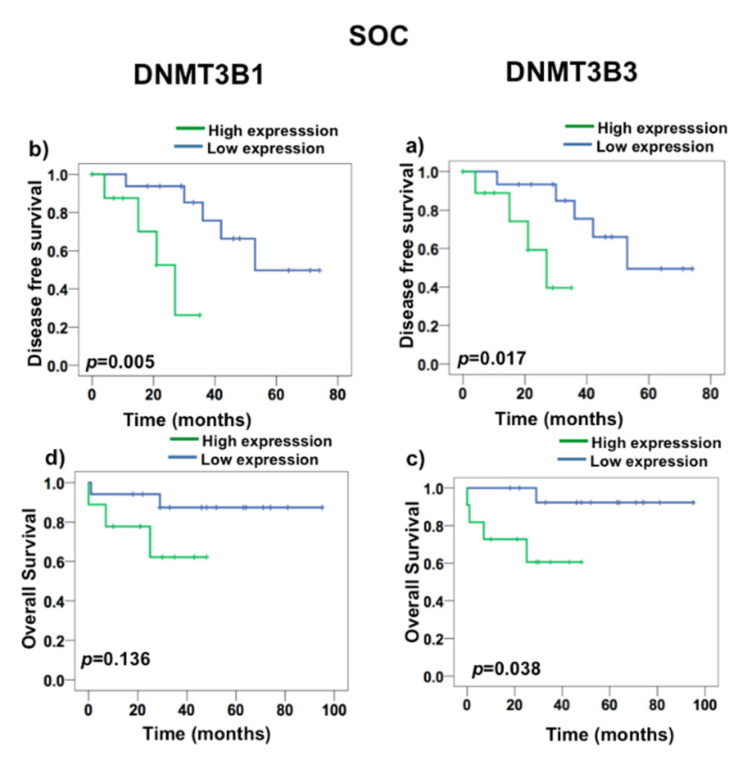
DNMT3B3 overexpression is associated with disease-free survival in ovarian carcinoma (SOC). DNMT3B1 expression in Kaplan–Meyer survival analysis in EOC: (**a**) DFS and (**b**) OS, and in SOC: (**c**) DFS and (**d**) OS. The log rank test was used to calculate the significant differences among overexpression (n = 46) versus no overexpression (n = 23) groups.

To test if DNMT3B1 and DNMT3B3 have a prognostic value in death and recurrence, we first realized a ROC curve to determine the value that we consider as overexpression. We found that DNMT3B3 had a cut-off 3.29 in a curve area of 72%, 87% of sensitivity, and 71% of specificity (Supplementary Figure S1). Nonetheless, DNMT3B1 had curve areas greater than 50%, but they were less than the DNMT3B3 curve area. To evaluate the prognostic value of DNMT3B1 and DNMT3B3 overexpression, we performed a Kaplan–Meyer survival analysis (Figure 5 and Figure 6). DNMT3B1 overexpression was associated with DFS (*p* = 0.0043), but it did not associate with OS (*p* = 0.182) (Figure 5a,c), and DNMT3B3 overexpression was associated with both DFS (*p* = 0.002) and OS (*p* = 0.017) in EOC (Figure 5c,d). Additionally, DNMT3B1 overexpression was associated with DFS (*p* = 0.005) and did not associate with OS (*p* = 0.136) (Figure 6a,c). Interestingly, DNMT3B3 overexpression was associated with DFS (*p* = 0.002) and OS (*p* = 0.017) in SOC (Figure 6c,d). These results suggest that the overexpression of the DNMT3B3 isoform could be helpful as a prognostic biomarker in EOC cancer patients, in contrast to DNMT paralogues and DNMT3B isoforms.

### 2.4. DNA Methyltransferase 3B3 Expression in High-Grade, Advanced Ovarian Carcinoma Cell Lines

**Figure 7:**
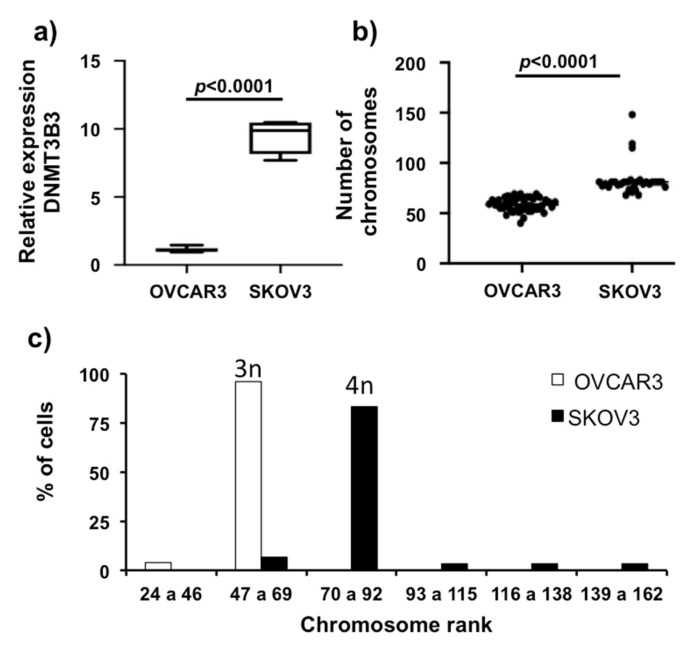
DNMT3B3 overexpression is associated with CIN in OVCAR3 and SKOV3 ovarian carcinoma cells. (**a**) DNMT3B3 mRNA expression by qRT-PCR, (**b**) number of chromosomes, and (**c**) frequency distribution of the number of chromosomes, according to the ploidy ranking, in advanced stages of high-grade serous ovarian carcinoma cell lines: OVCAR3 and SKOV3. The relative expression assay was normalized with GAPDH expression in each case and OSE cell lines as a control. Mann–Whitney U test was used.

We observed that ovarian cancer tumors have DNMT3B3 overexpression associated with poor prognosis of patients with ovarian carcinoma. Next, we validated the overexpression of DNMT3B3 in advanced, high-grade, serous ovarian carcinoma in cell lines. We measured the quantity of mRNA of DNMT3B3 from ovarian carcinoma cell lines OVCAR3 and SKOV3 compared to a primary culture: ovarian superficial epithelial (OSE). We found that OVCAR3 and SKOV3 overexpress DNMT3B3 compared with OSE (*p* < 0.001). SKOV3 overexpressed 10-fold the quantity of DNMT3B3 compared with OVCAR3 (*p* < 0.001) (Figure 7a). Next, we characterized chromosome numbers by counting cellular chromosomes (30 cellular metaphases) (Figure 7b,c). We found that these high-grade advanced ovarian carcinoma cell lines, OVCAR3 and SKOV3, have chromosome instability. The variation of chromosome numbers in OVCAR3 has a range from 40 to 69 chromosomes per cell (hipotriploid cell line) with a mode in 57 chromosomes (relative frequency of 14.58% of cells), and SKOV3 is more variable, with a range from 68 to 83 (hipotetraploid), with some cells present in lesser frequency: 115, 119, and 148 (relative frequency of 3.3% of cells) chromosomes per cell, having a mode in 81 chromosomes (relative frequency of 36.6% of cells). Interestingly, SKOV3 overexpresses DNMT3B3 and has more aggressive characters and a more variable number of chromosomes. These results suggest that DNMT3B3 overexpression could be a biomarker of tumor aggressiveness.

### 2.5. Overexpression of DNA Methyltransferase 3B3 Is a Cause of DNA Hypomethylation in the OVCAR3 Cell Line

**Figure 8:**
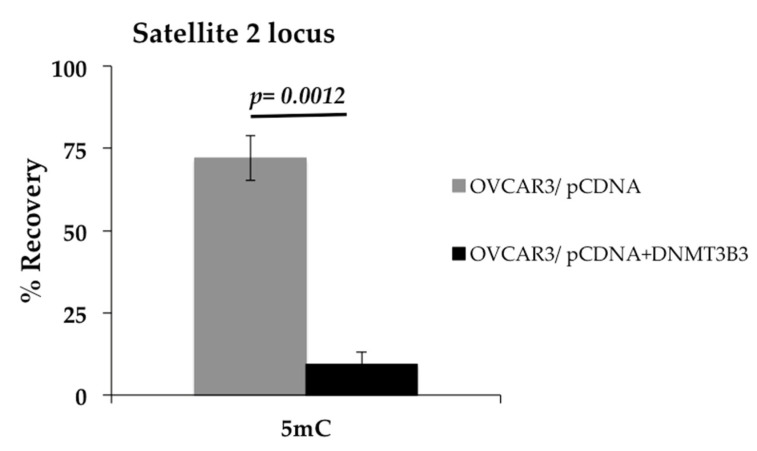
DNMT3B3 overexpression induces DNA hypomethylation in OVCAR3. Immunoprecipitation of 5′ methylcytosine was used to measure DNA methylation status in the satellite 2 locus: 15% of DNA methylation of the satellite 2 locus in OVCAR3 was detected after transfection of DNMT3B3. Mann–Whitney U test was used.

Since we observed an association of DNMT3B3 with poor prognosis in patients, we wanted to know if this could be related to DNA methylation of satellite 2, which is well-known as a possible cause of chromosome instability (CIN) [7]. Therefore, we analyzed the effect of exogenous overexpression of DNMT3B3 by transitory transfection in the OVCAR3 cell line. Then, we asked if overexpression of DNMT3B3 is a cause of DNA hypomethylation. Hence, we overexpressed DNMT3B3 and found that satellite 2 sequences were hypomethylated after the DNMT3B3 transfection in OVCAR3 (Figure 8).

## 3. Discussion

Our study has found that DNMT3B3 may be involved in EOC development and aggressiveness and suggested that some DNMT3B isoforms, such as DNMT3B3, could be used as a potential prognostic biomarker in EOC.

DNA methylation is an essential epigenetic marker to maintain genome stability and regulate DNA transcription [4]. Overexpression of DNMTs has been associated with hypermethylated tumor suppressor genes in cancer [38,39,40]. In addition, DNMT3B4 and DNMT3B7 isoforms’ overexpression has been suggested to trigger DNA hypomethylation [7,41,42]. DNMT3B has many isoform products of alternative splicing reported. It has been demonstrated that there are fifteen isoforms of DNMT3B [24]. The main characteristic in DNMT3B isoforms is the partial or total absence of the catalytic domain [22].

Previous studies have identified DNMT3B and DNMT3B3 overexpression in HGSOC cell lines (CAOV and SKOV3) by PCR [38]. In this study, we analyzed the overexpression of DNMTs in a cohort of EOC patients that included the histological subtypes of EOC, as well as patients in all the disease stages and the majority of patients with optimal cytoreduction surgery. Although the most frequent subtype was serous, we found interesting results that comprise all subtypes of EOC. We found the overexpression of DNMT3B isoforms: DNMT3B3, DNMT3B5, DNMT3B6, and DNMT3B3Δ5, in high-grade (G2 and G3) and advanced stages (III and IV). This result suggests a role of DNMT3B isoforms’ overexpression in the progression and aggressiveness of ovarian carcinoma. In SOC, we found that DNMT3B3 was overexpressed in high-grade and advanced stages. While in other EOC subtypes, we did not find significant differences. Then, we tried to confirm our hypothesis using commercial Ovarian Cancer cDNA arrays, where we also observed that DNMT3B3 is overexpressed in advanced stages of HGSOC. Consistent with our results, DNMT3B3 has been reported as overexpressed in gastric cancer, cancer cell lines derived from the brain, and cancer stem cells (293 cell line- derived from kidney embryo [19,23,24,33,40].

Ostler et al. have suggested that the abnormal DNA methylation patterns presented by cancer cells may be regulated by the enzymatically inactive DNMTB protein levels [19]. In the present study, we found that the overexpression of DNMT3B was associated with differentiation grades and stages of disease; specifically, DNMT3B3 was associated with disease progression, low DFS, and notably with OS in EOC. Furthermore, in agreement with our results, other studies have also reported the overexpression of DNMT3B3 in gastric cancer patients [33] and a correlation between DNMT3B3 expression and DNA hypomethylation in liver cancer cells [35].

Interestingly, the structure of DNMT3B3 possesses methyltransferase motifs I, IV, VI, IX, and X, suggesting that it has a potential methyltransferase activity, in contrast to DNMT3B4, where most of the methyltransferase motifs are missing.. [27]. In this sense, we found that overexpression of DNMT3B3 in the OVCAR3 cell line induces hypomethylation of DNA in the locus of satellite 2. Similar to our results, it has been reported that overexpression of DNMT3B4 causes hypomethylation on pericentromeric satellite regions in the epithelial 293 cell line [35]. Besides, it has been observed that DNMT3B isoforms compete with each other; for example, DNMT3B3 competes with DNMT3B4 to target DNA regions in human epithelial cancer cells [26]. Therefore, it could be possible that DNMT3B3 modulates DNA methylation in cancer, favoring DNA hypomethylation due to competition with DNMT3B1. In addition, the increased expression of DNMT3B1 and DNMT3B3 may induce a competence between active and inactive DNMT3B isoforms that results in higher aggressiveness and progression of EOC. To our knowledge, this is the first study that associates DNMT3B3 overexpression with poor prognosis in DFS and OS of EOC. These findings have led to a special interest in DNMT3B3 expression analysis in future research.

In conclusion, our results suggest that DNMT3B isoforms are abnormally expressed in OC and that DNMT3B3 overexpression may be involved in EOC development and aggressiveness. Finally, we suggest that DNMT3B3 could be used as a clinical prognostic biomarker in EOC.

## 4. Materials and Methods

**Tissue sample:** 63 tumors from patients with EOC and 10 non-cancerous ovarian samples were obtained at the National Cancer Institute, Mexico, from April 2011 to March 2018. The clinical–pathological characteristics of patients are shown in Table 1. The internal Review Board of the INCAN approved this project and the informed consent in 2011 (Register Number: 008/004/IBI).

**Reverse-transcription.** Total RNA was isolated using TRIzol Reagent (Termofisher, Waltham, MA, USA) (cat. no. 15596018), and first-strand cDNA was converted from 1 μg of RNA in a total volume of 20 μL with random primers and the High-Capacity Reverse Transcription Kit (Termofisher, Waltham, MA, USA) (cat. no. 4368814), according to the manufacturer’s instructions.

**Splice variants-specific quantitative RT-PCR.** cDNA (prepared as previously described) was subjected to quantitative real-time polymerase chain reaction (qPCR) for DNMT1, 3A, 3B, and DNMT3B3, 3B4, 3B5, 3B6, 3B7, and 3B3Δ5 isoforms using a Master Mix 2X SYBR Green/ROX qPCR kit (Termofisher, Waltham, MA, USA) (cat. no. K0222) under the following cycling conditions: PCR reactions were performed in a 30 μL volume with SYBR Green Master Mix, cDNA samples equivalent to 50 ng, and 0.3 μM of each primer for 40 cycles at 60 °C using QuantStudio 3 (Termofisher, Waltham, MA, USA). To normalize reactions, GAPDH was used as endogenous gene expression. The primer set used was previously reported by Liu et al. [34] and is listed in Table 2. To normalize and compare primers, we calculated the primer efficiencies to verify that they were 100%. The relative level of each DNMT3B isoform among the samples was then calculated using the 2^−ΔΔCt^ method [43].

**cDNA samples of TissueScan Ovarian Cancer cDNA arrays II and III.** Testing was carried out in two arrays of rapid cDNA panels: II and III, of ovarian carcinoma (TissueScan Ovarian Cancer cDNA arrays II and III) (OriGene Technologies, Rockville, MD, USA) (cat. no. HORT102 and 103) (n = 82). Arrays were prepared by the manufacturer’s instructions, using the DNMT isoforms’ primers per plate.

**Cell Culture.** SKOV3 and OVCAR3 cells were obtained from ATCC. OVCAR3 cells were cultured in RPMI medium 1640 (GIBCO/Thermo Scientific, Waltham, MA, USA) (cat. no. 11875) supplemented with 20% fetal bovine serum. SKOV3 cells were cultured in McCoy’s 5A medium (ATCC, MNZ, VA USA) (cat. no. 30-2007) supplemented with 10% fetal bovine serum. Cells were maintained at 37 °C in a 5% CO_2_ atmosphere.

**DNMT3B3 transfection in OVCAR3 cell line.** OVCAR3 cells were transitory transfected in all experiments of transfection. We used Lipofectamine 3000 (Invitrogen/Termofisher, Waltham, MA, USA) (cat. no. L3000-015) according to the manufacturer’s instructions, with a plasmid pcDNA/Myc_DNMT3B3 (Addgene, MA, USA) (plasmid: 37546), and pCDNA3.1 as the empty vector condition [43]. Cells were allowed to grow for 2 days before transfection until 70% confluence. No selection was applied during the culture. Transfections were repeated independently. Overexpression of DNMT3B3 was verified after transfection by qRT-PCR.

**Metaphases counting.** OVCAR3 cells were cultured on 6-well plates (Corning Inc., NY, USA) (cat. no. 3513), transfected, and treated to count metaphases. Cells were treated for 30 min with 90 ng/mL of colcemid (KaryoMAX GIBCO, USA) (cat. No. 15210-040) to induce the arrest in metaphase, and then incubated for 30 min at 37 °C in hypotonic solution (75 mM of KCl). Cells were fixed within 3:1 methanol:acetic acid, followed by GTG banding (G-banding using trypsin and Giemsa). A total of 30 metaphase cells were analyzed at the 400–500 band resolution level. For each condition, a certified cytogeneticist evaluated the metaphases in duplicate.

**5-′Methyl DNA immunoprecipitation assay.** The assay was performed using the meDIP kit (Diagenode, MagMeDIP qPCR Kit, BE-WLG, and NJ, USA) (cat. no. C02010014) following the manufacturer’s instructions. OVCAR3 cells were cultured on 6-well plates (Corning Inc., USA) (cat. no. 3513) and transfected. Then, 1 × 10^6^ transfected cells were used to follow the manufacturer’s instructions. For meDIP analysis, we realized the amplification efficiency (AE) and used Ct^10% Input^ to obtain the % recovery = 2^^((Ctinput 10% − 3.32)−(CtIP sample) × 100)^. All qPCR reactions were performed by triplicate in a fast optical 96-well qPCR reaction plate (Applied Biosystems, USA) (cat. no. 4346907). We used the following primers: 5′-ATCGAATGGAAATGAAAGGAGTCA-3′ (forward) and 5′-GACCATTGGATGATTGCAGTCA-3′ (reverse) for human chromosome 1 juxtacentromeric satellite-2 (Abcam, ab85781).

**Statistical analysis**. Relative expression of DNMT1, 3A, 3B, and DNMT3B3, 3B4, 3B5, 3B6, 3B7, and 3B3Δ5 isoforms’ data was obtained by the 2^−ΔΔCt^ equation and transformed by log_10_ + 1; then, we obtained better normalized adjustment as a result. We used the Mann–Whitney U test to compare differences in clinical variables using Prism 8 software for Mac. The results of the DNMT1, 3A, 3B, and DNMT3B3, 3B4, 3B5, 3B6, 3B7, and 3B3Δ5 isoforms’ expression levels were shown by the Tukey test. ROC curve and Kaplan–Meyer analyses were calculated in SPSS 21 Mac software. We considered a statistically significant ROC curve area higher than 50% for each isoform expression in disease-free survival (DFS) and overall survival (OS). To perform Kaplan–Meyer survival analysis, we used the surgical date and recurrence date or last visit date to determine DFS and the surgical date and death date or last visit date to determine OS. We considered *p* < 0.05 statistically significant in the log rank probe from survival analysis.

## Figures and Tables

**Table 1:** Clinical characteristics of patients with ovarian carcinoma enrolled in this study.

Variable	Category	*n*	%
Stage	I	29	46
	II	7	11
	III	18	29
	IV	9	14
Histologic type	Serous	27	43
	Endometrioid	15	24
	Clear cells	11	17
	Mucinous	8	13
	Other	2	3
Tumor grade	G1	18	29
	G2	9	14
	G3	36	57
Recurrence	Yes	12	16
	No	51	84
Current final status	Dead	9	13
	Alive	54	87

Tumor grade: G1: low grade, G2: medium grade, G3: high grade.

**Table 2:** Primer sets.

Gene Name	Forward Primer	Reverse Primer
DNMT1	AACCTTCACCTAGCCCCAG	CTCATCCGATTTGGCTCTTTCA
DNMT3A	GACAAGAATGCCACCAAAGC	CCATCTCCGAACCACATGAC
DNMT3B1	CCATCAAAGTTTCTGCTGCT	GAAGAGGTGTCGGATGACAG
DNMT3B3	CCGGGATGAACAGGATCTTT	AGTAGTCCTTCAGAGGGGCG
DNMT3B4	CGGTTCCTGGAGTGTAATCC	GGTTATTGTCTGTACTTTCTTTAACTGTT
DNMT3B5	AATACAATAGGATAGCCAAGGATCT	TTCAGAGGGGCGAAGAGG
DNMT3B6	CCAAGCTTGGAAAGCATGAA	CCGTTGACGAGGATCGAGT
DNMT3B7	CAGTCTAATTACCTTTCACAGAGAACA	GCTTTGAGGCGCTTGGGT
DNMT3B3Δ5	GAAAGCCCAGCTTCCCTGAGA	AGTTGTGTCCTCTGTGTCGTCTGT
GAPDH	TCGTTCCCAAAGTCCTCCTGTTTC	TCCGCAGCCGCCTGGTTC

## Data Availability

Not applicable.

## Supplementary Materials

The supporting information can be downloaded at: https://www.mdpi.com/article/10.3390/ijms232112759/s1.

## References

[1] Bray F., Ferlay J., Soerjomataram I., Siegel R.L., Torre L.A., Jemal A. (2018). Global Cancer Statistics 2018: GLOBOCAN Estimates of Incidence and Mortality Worldwide for 36 Cancers in 185 Countries. CA Cancer J. Clin..

[2] Kurman R.J., Shih I.-M. (2010). The Origin and Pathogenesis of Epithelial Ovarian Cancer: A Proposed Unifying Theory. Am. J. Surg. Pathol..

[3] McCluggage W.G. (2011). Morphological Subtypes of Ovarian Carcinoma: A Review with Emphasis on New Developments and Pathogenesis. Pathology.

[4] Li E., Zhang Y. (2014). DNA Methylation in Mammals. Cold Spring Harb. Perspect. Biol..

[5] Jurkowska R.Z., Jurkowski T.P., Jeltsch A. (2011). Structure and Function of Mammalian DNA Methyltransferases. ChemBioChem.

[6] Vavouri T., Lehner B. (2012). Human Genes with CpG Island Promoters Have a Distinct Transcription-Associated Chromatin Organization. Genome Biol..

[7] Zeimet A.G., Fiegl H., Goebel G., Kopp F., Allasia C., Reimer D., Steppan I., Mueller-Holzner E., Ehrlich M., Marth C. (2011). DNA Ploidy, Nuclear Size, Proliferation Index and DNA-Hypomethylation in Ovarian Cancer. Gynecol. Oncol..

[8] Widschwendter M., Jiang G., Woods C., Müller H.M., Fiegl H., Goebel G., Marth C., Müller-Holzner E., Zeimet A.G., Laird P.W. (2004). DNA Hypomethylation and Ovarian Cancer Biology. Cancer Res..

[9] Wiley A., Katsaros D., Chen H., Rigault de la Longrais I.A., Beeghly A., Puopolo M., Singal R., Zhang Y., Amoako A., Zelterman D. (2006). Aberrant Promoter Methylation of Multiple Genes in Malignant Ovarian Tumors and in Ovarian Tumors with Low Malignant Potential. Cancer.

[10] Shao C., Lacey M., Dubeau L., Ehrlich M. (2009). Hemimethylation Footprints of DNA Demethylation in Cancer. Epigenetics.

[11] Press J.Z., De Luca A., Boyd N., Young S., Troussard A., Ridge Y., Kaurah P., Kalloger S.E., Blood K.A., Smith M. (2008). Ovarian Carcinomas with Genetic and Epigenetic BRCA1 Loss Have Distinct Molecular Abnormalities. BMC Cancer.

[12] De Caceres I.I., Battagli C., Esteller M., Herman J.G., Dulaimi E., Edelson M.I., Bergman C., Ehya H., Eisenberg B.L., Cairns P. (2004). Tumor Cell-Specific BRCA1 and RASSF1A Hypermethylation in Serum, Plasma, and Peritoneal Fluid from Ovarian Cancer Patients. Cancer Res..

[13] Ozdemir F., Altinisik J., Karateke A., Coksuer H., Buyru N. (2012). Methylation of Tumor Suppressor Genes in Ovarian Cancer. Exp. Ther. Med..

[14] Moselhy S.S., Kumosani T.A., Kamal I., Jalal J., Jabaar H.S.A., Dalol A. (2015). Hypermethylation of P _15_, P _16_, and E-Cadherin Genes in Ovarian Cancer. Toxicol. Ind. Health.

[15] Chédin F. (2011). The DNMT3 Family of Mammalian De Novo DNA Methyltransferases. Progress in Molecular Biology and Translational Science.

[16] Okano M., Bell D.W., Haber D.A., Li E. (1999). DNA Methyltransferases Dnmt3a and Dnmt3b Are Essential for De Novo Methylation and Mammalian Development. Cell.

[17] Ratnam S., Mertineit C., Ding F., Howell C.Y., Clarke H.J., Bestor T.H., Chaillet J.R., Trasler J.M. (2002). Dynamics of Dnmt1 Methyltransferase Expression and Intracellular Localization during Oogenesis and Preimplantation Development. Dev. Biol..

[18] Manzo M., Wirz J., Ambrosi C., Villaseñor R., Roschitzki B., Baubec T. (2017). Isoform-specific Localization of DNMT3A Regulates DNA Methylation Fidelity at Bivalent CpG Islands. EMBO J..

[19] Ostler K.R., Davis E.M., Payne S.L., Gosalia B.B., Expósito-Céspedes J., Le Beau M.M., Godley L.A. (2007). Cancer Cells Express Aberrant DNMT3B Transcripts Encoding Truncated Proteins. Oncogene.

[20] Ma M.Z., Lin R., Carrillo J., Bhutani M., Pathak A., Ren H., Li Y., Song J., Mao L. (2015). ∆ DNMT3B4-Del Contributes to Aberrant DNA Methylation Patterns in Lung Tumorigenesis. EBioMedicine.

[21] Wang J., Bhutani M., Pathak A.K., Lang W., Ren H., Jelinek J., He R., Shen L., Issa J.-P., Mao L. (2007). *ΔDNMT3B* Variants Regulate DNA Methylation in a Promoter-Specific Manner. Cancer Res..

[22] Wang L., Wang J., Sun S., Rodriguez M., Yue P., Jang S., Mao L. (2006). A Novel DNMT3B Subfamily, ΔDNMT3B, Is the Predominant Form of DNMT3B in Non-Small Cell Lung Cancer. Int. J. Oncol..

[23] Gopalakrishnan S., Van Emburgh B.O., Shan J., Su Z., Fields C.R., Vieweg J., Hamazaki T., Schwartz P.H., Terada N., Robertson K.D. (2009). A Novel DNMT3B Splice Variant Expressed in Tumor and Pluripotent Cells Modulates Genomic DNA Methylation Patterns and Displays Altered DNA Binding. Mol. Cancer Res..

[24] Okano M., Xie S., Li E. (1998). Cloning and Characterization of a Family of Novel Mammalian DNA (Cytosine-5) Methyltransferases. Nat. Genet..

[25] Weisenberger D.J., Velicescu M., Cheng J.C., Gonzales F.A., Liang G., Jones P.A. (2004). Role of the DNA Methyltransferase Variant DNMT3b3 in DNA Methylation. Mol. Cancer Res..

[26] Aoki A. (2001). Enzymatic Properties of de Novo-Type Mouse DNA (Cytosine-5) Methyltransferases. Nucleic Acids Res..

[27] Gordon C.A., Hartono S.R., Chédin F. (2013). Inactive DNMT3B Splice Variants Modulate De Novo DNA Methylation. PLoS ONE.

[28] Shao G., Zhang R., Zhang S., Jiang S., Liu Y., Zhang W., Zhang Y., Li J., Gong K., Hu X.-R. (2013). Splice Variants DNMT3B4 and DNMT3B7 Overexpression Inhibit Cell Proliferation in 293A Cell Line. Vitro Cell. Dev. Biol. Anim..

[29] Ostler K.R., Yang Q., Looney T.J., Zhang L., Vasanthakumar A., Tian Y., Kocherginsky M., Raimondi S.L., DeMaio J.G., Salwen H.R. (2012). Truncated DNMT3B Isoform DNMT3B7 Suppresses Growth, Induces Differentiation, and Alters DNA Methylation in Human Neuroblastoma. Cancer Res..

[30] Gopalakrishna-Pillai S., Iverson L.E. (2011). A DNMT3B Alternatively Spliced Exon and Encoded Peptide Are Novel Biomarkers of Human Pluripotent Stem Cells. PLoS ONE.

[31] Plourde K.V., Labrie Y., Ouellette G., Pouliot M.-C., Durocher F. (2016). Genome-Wide Methylation Analysis of *DNMT3B* Gene Isoforms Revealed Specific Methylation Profiles in Breast Cell Lines. Epigenomics.

[32] Duymich C.E., Charlet J., Yang X., Jones P.A., Liang G. (2016). DNMT3B Isoforms without Catalytic Activity Stimulate Gene Body Methylation as Accessory Proteins in Somatic Cells. Nat. Commun..

[33] Su X., Lv C., Qiao F., Qiu X., Huang W., Wu Q., Zhao Z., Fan H. (2010). Expression Pattern and Clinical Significance of DNA Methyltransferase 3B Variants in Gastric Carcinoma. Oncol. Rep..

[34] Liu Y., Sun L., Fong P., Yang J., Zhang Z., Yin S., Jiang S., Liu X., Ju H., Huang L. (2017). An Association between Overexpression of DNA Methyltransferase 3B4 and Clear Cell Renal Cell Carcinoma. Oncotarget.

[35] Saito Y., Kanai Y., Sakamoto M., Saito H., Ishii H., Hirohashi S. (2002). Overexpression of a Splice Variant of DNA Methyltransferase 3b, DNMT3b4, Associated with DNA Hypomethylation on Pericentromeric Satellite Regions during Human Hepatocarcinogenesis. Proc. Natl. Acad. Sci. USA.

[36] Brambert P.R., Kelpsch D.J., Hameed R., Desai C.V., Calafiore G., Godley L.A., Raimondi S.L. (2015). DNMT3B7 Expression Promotes Tumor Progression to a More Aggressive Phenotype in Breast Cancer Cells. PLoS ONE.

[37] Siddiqui S., White M.W., Schroeder A.M., DeLuca N.V., Leszczynski A.L., Raimondi S.L. (2018). Aberrant DNMT3B7 Expression Correlates to Tissue Type, Stage, and Survival across Cancers. PLoS ONE.

[38] Ahluwalia A., Hurteau J.A., Bigsby R.M., Nephew K.P. (2001). DNA Methylation in Ovarian Cancer: II. Expression of DNA Methyltransferases in Ovarian Cancer Cell Lines and Normal Ovarian Epithelial Cells. Gynecol. Oncol..

[39] Van Emburgh B.O., Robertson K.D. (2011). Modulation of Dnmt3b Function in Vitro by Interactions with Dnmt3L, Dnmt3a and Dnmt3b Splice Variants. Nucleic Acids Res..

[40] Robertson K.D., Uzvolgyi E., Liang G., Talmadge C., Sumegi J., Gonzales F.A., Jones P.A. (1999). The Human DNA Methyltransferases (DNMTs) 1, 3a and 3b: Coordinate MRNA Expression in Normal Tissues and Overexpression in Tumors. Nucleic Acids Res..

[41] Wang J., Walsh G., Liu D.D., Lee J.J., Mao L. (2006). Expression of *ΔDNMT3B* Variants and Its Association with Promoter Methylation of *P16* and *RASSF1A* in Primary Non–Small Cell Lung Cancer. Cancer Res..

[42] Ehrlich M., Woods C.B., Yu M.C., Dubeau L., Yang F., Campan M., Weisenberger D.J., Long T., Youn B., Fiala E.S. (2006). Quantitative Analysis of Associations between DNA Hypermethylation, Hypomethylation, and DNMT RNA Levels in Ovarian Tumors. Oncogene.

[43] Livak K.J., Schmittgen T.D. (2001). Analysis of Relative Gene Expression Data Using Real-Time Quantitative PCR and the 2−ΔΔCT Method. Methods.

