# Supplementary material for: Expression of DNA Methyltransferase 3B Isoforms Is Associated with DNA Satellite 2 Hypomethylation and Clinical Prognosis in Advanced High-Grade Serous Ovarian Carcinoma
Source: Int J Mol Sci. 2022 Oct 22;23(21):12759. doi: 10.3390/ijms232112759 (PMC9654283; doi:10.3390/ijms232112759)
Supplement: Supplementary file 1 [file ijms-23-12759-s001.zip › ijms-1899708-supplementary.pdf]

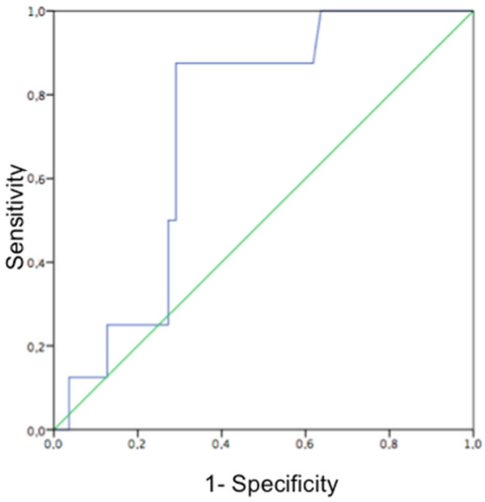

Supplementary Figure S1. DNMT3B3 is associated with poor prognostic in EOC. DNMT3B3 ROC curve of EOC deaths. The curve area was 0.705, with a cut value calculated by the ROC curve was 3.29 with 85% of sensitivity and 66% of specificity. 36% of patients in this study overexpress DNMT3B3 and could have poor clinical prognostic.
